# Age-related hearing loss is associated with alterations in temporal envelope processing in different neural generators along the auditory pathway

**DOI:** 10.3389/fneur.2022.905017

**Published:** 2022-08-05

**Authors:** Ehsan Darestani Farahani, Jan Wouters, Astrid van Wieringen

**Affiliations:** Research Group Experimental ORL, Department Neurosciences, KU Leuven, Leuven, Belgium

**Keywords:** age-related hearing loss (ARHL), neural generators, auditory temporal processing, auditory steady-state response (ASSR), EEG

## Abstract

People with age-related hearing loss suffer from speech understanding difficulties, even after correcting for differences in hearing audibility. These problems are not only attributed to deficits in audibility but are also associated with changes in central temporal processing. The goal of this study is to obtain an understanding of potential alterations in temporal envelope processing for middle-aged and older persons with and without hearing impairment. The time series of activity of subcortical and cortical neural generators was reconstructed using a minimum-norm imaging technique. This novel technique allows for reconstructing a wide range of neural generators with minimal prior assumptions regarding the number and location of the generators. The results indicated that the response strength and phase coherence of middle-aged participants with hearing impairment (HI) were larger than for normal-hearing (NH) ones. In contrast, for the older participants, a significantly smaller response strength and phase coherence were observed in the participants with HI than the NH ones for most modulation frequencies. Hemispheric asymmetry in the response strength was also altered in middle-aged and older participants with hearing impairment and showed asymmetry toward the right hemisphere. Our brain source analyses show that age-related hearing loss is accompanied by changes in the temporal envelope processing, although the nature of these changes varies with age.

## Introduction

Speech perception of individuals with hearing impairment (HI) is worse than that of persons with normal audiometric thresholds (NH), even after correcting for differences in hearing audibility (1–4). In addition to deficits in audibility, changes in central auditory processing, and in particular temporal processing, account for impaired speech perception of individuals with HI (5). Electrophysiological studies in animals have shown that HI is associated with increased neural responses to amplitude-modulated stimuli in the auditory nerve fibers (6–8) and the midbrain (9). Similarly, human studies showed enhanced neural responses in the brainstem of adults around 60 years old with HI compared to NH ones in the same age range (10, 11).

The temporal envelope of speech (slow fluctuations of 2 to 50 Hz) is crucial for accurate speech understanding (12–14) and transmits both prosodic and linguistic information (15). Speech envelopes are encoded in the central auditory system through synchronized (phase-locked) neural activity (16, 17). Temporal envelope processing can be assessed through the auditory steady-state responses (ASSRs; 16). ASSRs are auditory-evoked responses to periodically varying acoustic stimuli and reflect the ability of the auditory system to follow the temporal envelope of sounds (18).

In our previous study (19), we investigated age-related changes in the activity of subcortical and cortical neural generators of ASSRs in middle-aged and older persons with normal audiometric thresholds (<25 dB HL). Analyses showed enhanced neural responses for older adults compared to younger ones for relatively slow modulations (<50 Hz). However, for faster modulations (i.e., 80 Hz), the neural responses were reduced for older adults compared to younger ones. While these age-related changes occur in persons with normal hearing, it remains unclear how HI affects temporal envelope processing. Aging is typically accompanied by decreasing audiometric thresholds in the high frequencies (presbycusis). These peripheral changes are accompanied by changes in the central auditory system (10, 20) and associated neural generators. The current study focuses on the potential aggravating role of HI on the activity of the neural generators for middle-aged and older adults.

Electrophysiological studies investigating how HI affects the processing of the temporal envelope demonstrated enhanced response strengths for middle-aged listeners with HI compared to middle-aged NH ones [~60 years old; (10, 11, 21, 22)]. In contrast to middle-aged persons with HI, older adults with HI (~75 years old) did not show enhanced responses to acoustic modulations (11). Note that stimulus audibility has been corrected in these studies. The absence of an enhanced response in older persons with HI could be because a significant neural enhancement had already been observed with NH older listeners and was, therefore, more a factor of aging than HI. However, how HI affects the temporal envelope processing in the different neural generators in middle-aged and older adults remains unclear. Sensor-level analysis (i.e., analysis based on the scalp's data) may not be sensitive enough to reveal all the dynamics of the neural generators underlying temporal envelope processing in persons with HI. This is because the recorded data at each sensor are a weighted average of the activity of several neural generators due to the volume conduction of the brain tissue.

On the other hand, brain source analysis estimates the original activity of each neural generator using computational modeling. In the current study, we use a source reconstruction approach based on minimum-norm imaging (MNI). In this approach, a large number of equivalent current dipoles in the brain are considered. Then, the amplitudes of all dipoles (for each time point) are estimated to reconstruct a source distribution map with minimum overall energy (23, 24).

The MNI approach imposes minimal restrictions about the number and location of the sources, contrary to more common methods like dipole source analysis, which makes prior assumptions regarding the number and location of the sources. Another advantage of the MNI approach is the ability to reconstruct a wide range of cortical and subcortical sources simultaneously (25). The beamforming method, another well-known method of brain source reconstruction, has more difficulty in reconstructing the cortical and subcortical sources. To reconstruct neural generators of ASSRs using beamforming methods, a supplementary preprocessing is necessary to suppress the correlated source from the other hemisphere (26–28). Additionally, the beamforming approaches cannot simultaneously reconstruct the cortical and subcortical sources.

Age-related hearing loss may also affect hemispheric asymmetry in temporal envelope processing. Previous data have shown that the pattern of neural synchronization in older adults with normal audiometric thresholds is symmetrical across hemispheres, while that of young NH adults is asymmetric (29, 30). With age, this altered hemispheric asymmetry is in line with the HAROLD model (31), which states that hemispheric asymmetry is reduced in older people compared to younger ones. Using brain source analyses, Farahani et al. (19) also showed that hemispheric asymmetry is reduced for NH older adults compared to younger normal hearing in response to the 20 and 80 Hz amplitude-modulated stimuli. However, age-related hearing loss may affect hemispheric asymmetry on top of age, as has been demonstrated for linguistic processing (32). In their sensor-level EEG study, Goossens et al. (11) observed a hemispheric asymmetry toward the right hemisphere for older participants with HI. The observed changes in hemispheric asymmetry in persons with HI are possibly due to anatomical changes related to presbycusis, such as reduced integrity of white matter tracts (33). However, it is also possible that the sensor-level analysis cannot capture changes related to HI in the other cohorts. It is expected that source-level analysis, due to the higher sensitivity explained before, might reflect more changes associated with HI concerning the hemispheric asymmetry than the sensor-level analysis.

The current study aims to investigate potential changes in temporal envelope processing for subcortical and cortical neural generators along the auditory pathway in middle-aged and older persons with age-related HI compared to normal-hearing ones. Different studies have shown that the diminished cochlear output of people with HI, due to hair cell loss and/or synaptopathy, activates various mechanisms to increase central gain and preserve neural excitability (e.g., 32, 33). Hence, we hypothesize that the neural generators of ASSRs in middle-aged listeners with HI will show enhanced response strength compared to those with NH. However, we do not expect such an enhancement in older adults with HI because older adults with NH already exhibit compensatory mechanisms of increasing neural excitability and central gain (19, 34, 35). Concerning hemispheric asymmetry in temporal envelope processing, we hypothesize that the reconstructed activity at the auditory cortex reveals an altered pattern of hemispheric asymmetry in listeners with HI. However, these alterations may vary with age and stimulation conditions.

We investigate the potential alterations during temporal envelope processing of people with HI when stimulus audibility was corrected for. We look into ASSRs' cortical and subcortical neural generators along the auditory pathway in young, middle-aged, and older persons with and without HI. The activity of these neural generators is reconstructed using a minimum-norm imaging (MNI) approach (25). To investigate the response strength and the phase-locking to the stimulus, the ASSR amplitude and phase coherence are calculated for each neural generator. This is done for ASSRs in response to 4, 20, 40, and 80 Hz acoustic modulations presented separately to the left and right ears. The acoustic modulations at 4 and 20 Hz were presented as a model of the temporal envelope of syllables and phonemes, respectively. The modulation frequencies of 40 and 80 Hz were also selected because these modulations can activate more subcortical neural generators than cortical ones (26, 36). Potential alterations in hemispheric asymmetry are also investigated for the neural generators in the left and right auditory cortices (31, 37).

## Materials and Methods

### Participants

**Figure 1:**
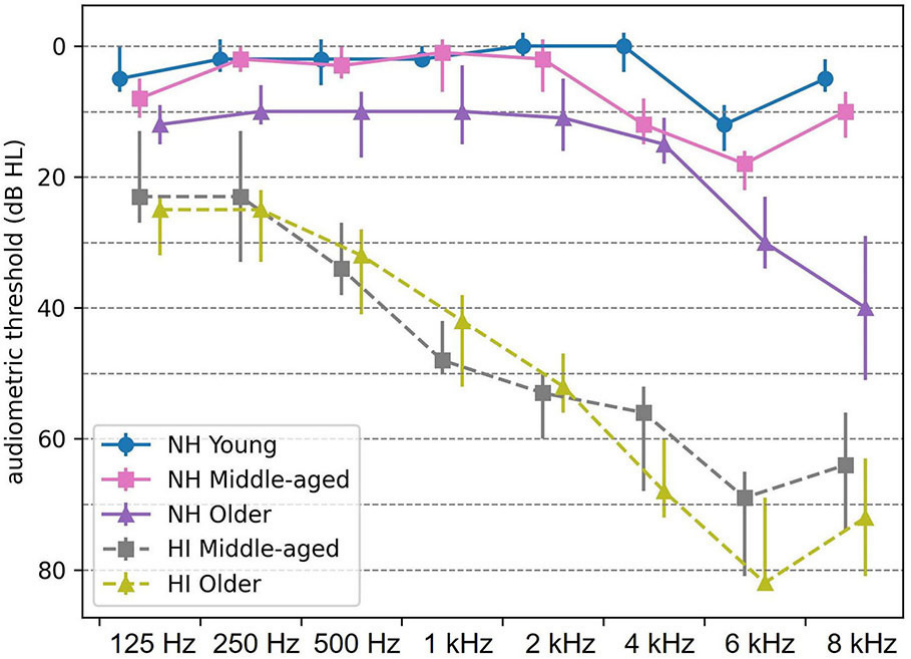
Median audiometric thresholds (dB HL) of normal-hearing (NH) and hearing-impaired (HI) participants, averaged across both ears. Thresholds are indicated by circles, squares, and triangles for young, middle-aged, and older persons, respectively. Error bars indicate the interquartile range. NH, normal hearing; HI, hearing impaired.

The EEG data were adopted from Goossens et al. (29). Participants were either NH or with HI in three narrow age cohorts, including 19 young (20–30 years, nine men), 20 middle-aged (50–60 years, ten men), and 16 older adults (70–80 years, five men) in NH group and 14 middle-aged (50–60 years, four men) and 13 older adults (70–80 years, five men) with HI. Only individuals who showed symmetrical hearing based on the criteria of the audiogram classification system (38) were eligible for participation. The participants in the NH group had audiometric thresholds within normal limits [ ≤ 25 dB HL] at all octave frequencies from 125 Hz up to and including 4 kHz in both ears (Figure 1). However, the participants with HI had audiometric thresholds higher than 35 dB HL from 1 kHz onward (Figure 1). All middle-aged and older participants with HI were diagnosed with age-related hearing loss (i.e., presbycusis) and used hearing aids in both ears. To avoid cognitive impairment as a confounder, only adults who showed no indication of cognitive impairment were recruited. The participants were screened using the Montreal Cognitive Assessment Task (39), and the cutoff score was 26 out of 30. This screening with a stringent cutoff score ensured that all participants had cognitive capacities within the normal range. All participants were Dutch native speakers. They were right-handed based on the Edinburgh Handedness Inventory (40), and none of them had a medical history of brain injury, neurological disorders, or tinnitus.

### Stimuli

The acoustic stimuli were amplitude-modulated (AM) noise at 4, 20, 40, and 80 Hz and generated in MATLAB (The MathWorks, Inc.). The white noise (bandwidth of 1 octave, centered at 1 kHz) was sinusoidally modulated with a modulation depth of 100%. The modulation frequencies were adjusted to ensure that there was an integer number of cycles in an epoch of 1.024 s (41).

#### Loudness balancing

The stimuli were presented *via* ER-3A insert phones to the left ear and the right ear. Each stimulus type was presented for 300 s continuously. For NH participants, the stimuli were presented at 70 dB SPL which they rated as comfortably loud. For participants with HI, no hearing aids were used during EEG recording. To correct for the audibility of listeners with HI, each individual was asked to adjust the intensity level until he/she perceived it as comfortably loud, similar to the NH participants. This arrangement allowed us to present stimuli to all participants at equal loudness levels. There were two reasons for using equal loudness levels to correct for stimulus audibility instead of equal sensation levels. First, the equal sensation level for participants with HI reaches ~108 dB SPL, which exceeds their uncomfortable loudness level (~103 dB SPL). Second, it was shown that the magnitude of the ASSR was highly correlated with the perceived loudness of the acoustic modulations (42, 43). So, the equal loudness level is an effective way to control for differences in stimulus audibility between NH and HI.

### Experiment protocol and EEG recordings

The experiment was conducted in a double-walled soundproof booth with a Faraday cage. The experiment procedure was arranged to ensure passive listening to acoustic stimuli during a wakeful state. During acoustic stimulation, the participants were asked to lay down on a bed and watch a muted movie with subtitles *via* a 21-inch LCD monitor with 60 Hz vertical refresh rate. All participants were encouraged to lie quietly and relaxed during the experiment to avoid movements and muscle artifacts caused by fatigue, especially in older adults. We used a large-size and very soft pillow to support the neck and backside of the head.

The EEG data were recorded using the BioSemi ActiveTwo system (BioSemi B.V., Amsterdam, the Netherlands, 2010) with 64 active electrodes. The electrodes were fixed in a head cap according to the 10–10 electrode system. The EEG signals were amplified and digitized at a sampling rate of 8,192 Hz with a gain of 32.25 nV/bit. The recording system used a built-in low-pass filter with a cutoff frequency of 1,638 Hz.

### EEG source analysis

The activity of the neural generators of ASSRs along the auditory pathway was reconstructed using a method based on MNI, which was suggested for ASSR source analysis (25). An overview of this method is given below [for more details, see (25)]. The analyses were performed in MATLAB R2016b (MathWorks).

#### Preprocessing

To eliminate the low-frequency distortions and drift of the amplifier, the EEG data were filtered by a zero-phase high-pass filter with a cutoff frequency of 2 Hz (Butterworth, second order, 12 dB/octave). The filtered EEG data were split into epochs of 1.024 s. Subsequently, 10% of epochs with the highest peak-to-peak amplitude across channels were rejected for early noise reduction.

Afterward, the EEG data were re-referenced to a common average over all channels and epochs. To eliminate artifacts caused by eye movements, eye blinks, and heartbeats, we used independent component analysis (ICA) based on the Infomax algorithm implemented in the FieldTrip toolbox (44). The noisy components were identified with a visual inspection. In the end, the remaining artifacts not recognized by ICA were identified and eliminated using a threshold level of 70 μV for the maximum absolute amplitude of each epoch. To have a similar effect on the group-wise results, we kept the same number of epochs across participants. The first 192 artifact-free epochs (six sweeps of 32 epochs) were preserved for subsequent analyses to keep the same number of epochs across participants. We chose not to use a lower number of epochs in each sweep to keep our frequency resolution high enough (each frequency bin corresponds to 0.03 Hz). In case we could not find 192 epochs (six sweeps of 32 epochs) for a participant, then we gradually increased the threshold (step of 5 μV) up to 110 μV. These epochs were selected out of 300 epochs of each participant per condition. For the topographic map of ASSRs, see Farahani et al. (45).

#### Source reconstruction and developing ASSR map

##### Mixed head model

A mixed head model consisting of cortical and subcortical regions was generated to reconstruct the neural generators along the auditory pathway. This head model was generated using the boundary element method (BEM), as implemented in OpenMEEG (46). To this end, we used the template brain scan of ICBM152 (47) and the default channel location file in the Brainstorm application (48, 49).

##### Data averaging for group-wise analyses

Since the head model was generated based on a template brain scan, we used a group-wise framework in our source analyses instead of individual-level analyses to have a high localization accuracy (45). So, the preprocessed epochs of each participant were divided into sweeps of 32 concatenated epochs and averaged across all participants. The outcome grand-averaged sweep was used for source reconstruction.

##### Reconstruction source map of EEG in time domain

The distribution map of brain activity at each time point was estimated using dynamic statistical parametric mapping [dSPM; (50)] implemented in the Brainstorm application (48, 49). In the dSPM method, the standard minimum-norm solution is normalized with the estimated noise at each source (24). This noise normalization eliminates the bias toward superficial sources, which is accompanied by the standard minimum-norm solution (24, 51).

##### Noise covariance matrix

The noise covariance matrix required for dSPM was calculated based on the EEG recorded in the absence of auditory stimulation. The silence EEG of participants was filtered by a zero-phase band-pass filter with a bandwidth of 4 Hz and modulation frequency as center frequency and concatenated before calculating the covariance matrix.

##### Regularization parameter

For each experimental condition (i.e., stimulation type, age group, and hearing status), the regularization parameter (λ^2^) required for dSPM was specifically determined based on:

Equation 1


$$\lambda^{2}=\frac{1}{\text{SN}{\text{R}}_{\text{scalp}}^{2}}$$

where SNR_scalp_ is the signal-to-noise ratio (based on the amplitude) of the whitened EEG data (52–54). The fast Fourier transform (FFT) was applied for each channel, and the magnitude of the spectrum at the modulation frequency was considered the ASSR strength. The highest response magnitude across channels was assigned to the signal of interest (19). The EEG background noise was estimated based on the average magnitude of 30 neighboring frequency bins on the left and the right sides of the response frequency bin. The median of the EEG background noise across channels was used as noise level for calculating SNR_scalp_ (25).

##### Generating ASSR map

ASSR map shows the magnitude of the response for different regions of the brain. To generate an ASSR map, the waveform of each dipole was transformed to the frequency domain using FFT. Then, for each dipole, the SNR of the ASSR was calculated according to Equation 2.

Equation 2


$$\text{SNR}(\text{dB})=10\;\left(\frac{{\text{P}}_{\text{S}+\text{N}}}{{\text{P}}_{\text{N}}}\right)$$

where P_S+N_ is the power of the spectrum at the modulation frequency, which shows the power of the steady-state response plus neural background noise. P_N_ indicates the power of the neural background noise, which was estimated using the average power of 30 neighboring frequency bins (corresponding to 0.92 Hz) on each side of the modulation frequency bin.

The one-sample f-test based on SNR was employed to recognize the dipoles with significant ASSRs (43, 55). Results were corrected for multiple comparisons using the false discovery rate (FDR) method (56). Finally, the ASSR map illustrating ASSR amplitudes for dipoles with significant responses and zero for the dipoles with no significant responses was generated. The ASSR amplitude was calculated using Equation 3. For subcortical regions, the activity at each point was reconstructed using three orthogonal dipoles (across x, y, and z). The ASSR amplitude for subcortical regions was calculated based on Equation 4. A detailed explanation and a sample ASSR map can be retrieved from the study by Farahani et al. (25).

Equation 3


$$\text{ASS}{\text{R}}_{\text{amp}}=\sqrt{{\text{P}}_{\text{S}+\text{N}}}-\sqrt{{\text{P}}_{\text{N}}}$$

Equation 4


$$\text{Subcortical}\;\text{ASS}{\text{R}}_{\text{amp}}=\sqrt{ASSR_{amp\;x}^{2}+ASSR_{amp\;y}^{2}\;+\;ASSR_{amp\;z}^{2}}$$

##### Defining regions of interest

**Figure 2:**
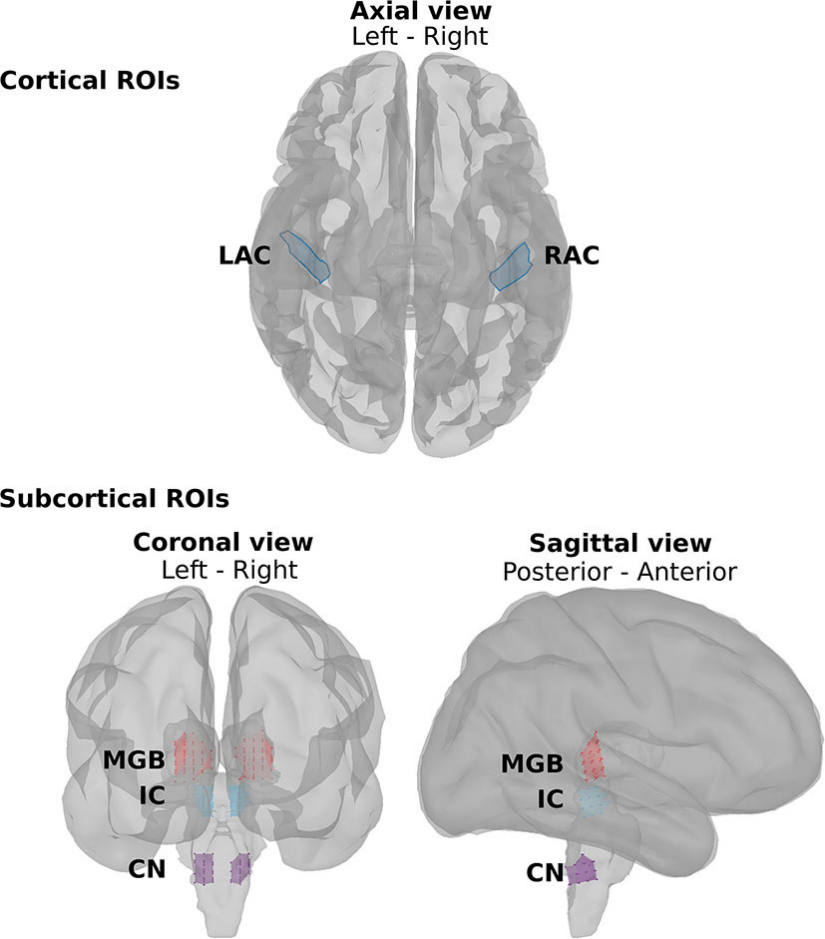
The regions of interest (ROIs) along the auditory pathway. The cortical ROIs are located bilaterally in the left auditory cortex (LAC) and right auditory cortex (RAC). The subcortical ROIs include the left and right medial geniculate body (LMGB, RMGB), the left and right inferior colliculus (LIC, RIC), and the left and right cochlear nucleus (LCN, RCN).

fMRI studies show that the main neural generators of the ASSRs along the auditory pathway are located in the cochlear nucleus (CN), the inferior colliculus (IC), the medial geniculate body (MGB), and the auditory cortex (AC) bilaterally (57–60). Therefore, we defined eight regions of interest (ROIs) for further analysis (Figure 2). At the subcortical level, the ROIs were defined bilaterally in the CN (recognized with reference to the medullary pontine junction; left CN: 0.49 cm^3^; right CN: 0.47 cm^3^), IC (identified with reference to the thalamus; left IC: 0.50 cm^3^; right IC: 0.55 cm^3^), and in the posterior thalamus (roughly the posterior third of the thalamus; left MGB: 1.24 cm^3^; right MGB: 1.45 cm^3^) (19, 60). The cortical ROIs of the AC were defined bilaterally in the Heschl's gyrus (left AC: 5.49 cm^2^; right AC: 5.58 cm^2^) with reference to the transverse temporal gyrus in the Desikan–Killiany atlas implemented in Brainstorm (48, 61).

##### Time series of ROIs and ASSR amplitude

A representative dipole in each ROI was selected for subsequent analysis using the algorithm suggested by Farahani et al. (25). First, inside each ROI, a patch with the highest mean ASSR amplitude was selected. Then, a dipole with the most similar response, regarding amplitude and phase, to the mean ASSR of the patch was selected as the representative dipole. The ASSR amplitudes of the representative dipoles in cortical and subcortical ROIs were obtained based on Eq. 3 and Eq. 4 and used for further analyses. The time series of the representative dipole was used for subsequent phase coherence analysis.

### Phase coherence

Phase coherence (or intertrial phase coherence) shows the phase consistency of ASSRs across epochs (17, 62). It also explains the phase-locking capability of a neural generator to the acoustic stimulus and varies between 0 and 1 (45, 63). To calculate the phase coherence, the time series of each ROI with 192 epochs were divided into 64 groups of three epochs. The phase of group i (θ_i_, i = 1, 2,..., 64) was obtained from the complex responses averaged across the three epochs. Finally, phase coherence was calculated based on Equation 5 (62).

Equation 5


$$\text{PhaseCoherence}=\frac{1}{\text{N}}\sqrt{{\left(\sum_{i=1}^{N}\text{cos}\theta_{i}\right)}^{2}+{\left(\sum_{i=1}^{N}\text{sin}\theta_{i}\right)}^{2}}$$

For subcortical ROIs, the representative dipole had three time series (x, y, and z components). To reduce the dimension of this data, the optimal dipole direction representing most of the variance of the ASSR was estimated using singular value decomposition (SVD) (64). The three time series were projected in the optimal direction, and the outcome was used for calculating the phase coherence. It should be noted that before SVD, the three time series were filtered by a zero-phase band-pass filter with a bandwidth of 4 Hz and modulation frequency as the center frequency.

### Hemispheric lateralization

To assess hemispheric asymmetry, we employed the laterality index (LI). The LI is a normalized index with the range of [-1, 1], where zero means symmetrical processing pattern and positive and negative values show lateralization to the right and left hemispheres, respectively. LI was calculated as:

Equation 6


$$\text{LI}=\frac{\text{ASS}{\text{R}}_{\text{amp}\;\text{R}}-\text{ASS}{\text{R}}_{\text{amp}\;\text{L}}}{\text{ASS}{\text{R}}_{\text{amp}\;\text{R}}+\text{ASS}{\text{R}}_{\text{amp}\;\text{L}}}$$

where ASSR_ampR_ and ASSR_ampL_ denote the ASSR amplitude (based on equations 3 and 4) of the neural generator located in the right and left hemispheres, respectively. To prevent inaccurate lateralization, the LI was only calculated when both neural generators had a significant ASSR.

### Statistical analysis

Since we used a group-wise framework and the value of ASSR measures could not be obtained for each individual participant, the standard deviation could not be calculated in the traditional manner. The standard deviation was estimated based on the jackknife resampling method for each of the ASSR amplitude, phase coherence, and LI (65). The mean of ASSR amplitudes, phase coherence, and LI were obtained from all participants without resampling. The subsequent statistical analyses were performed based on the mean, estimated standard deviation, and the number of participants in each group, rather than on individual data points (66, 67) using custom scripts in MATLAB R2016b (MathWorks).

To investigate the overall effect of hearing impairment on ASSR amplitude, a factorial mixed analysis of variance (FM-ANOVA) with side of stimulation (two levels: left and right) and neural generators (eight levels: two cortical generators and six subcortical generators) as within-subject variables was separately carried out for middle-aged and older participants in response to 4, 20, 40, and 80 Hz acoustic modulations. *Post-hoc* comparisons were performed in cortical and subcortical categories of neural generators. The two-sample *t*-test was performed for each category based on the pooled mean and the pooled standard deviations across neural generators. The results were corrected for multiple comparisons using the FDR method (56). In the tests with neural generators as a within-subject variable, the sample size of the test has a high number, and in turn, the statistical tests often showed very small *p*-values. Thus, the effect sizes were also reported to measure significance independent of sample size (68). Cohen's d was used as a measure of effect size. The description of magnitudes of d was initially suggested by Cohen (69) and expanded by Sawilowsky (70). The magnitudes of 0.01, 0.2, 0.5, 0.8, and 1.2 were described as very small, small, medium, large, and very large effect sizes. Similar statistical analyses were also carried out for phase coherence.

For hemispheric lateralization, a one-sample *t*-test with FDR correction was employed to determine for which stimulation conditions the LI differed significantly from zero. A significant positive or negative LI shows lateralization to the right or left hemispheres, respectively. Finally, the potential effect of hearing impairment on hemispheric lateralization was investigated using a two-sample *t*-test per modulation frequency and side of stimulation.

## Results

### Effect of hearing impairment on the response strength of the neural generators

**Figure 3:**
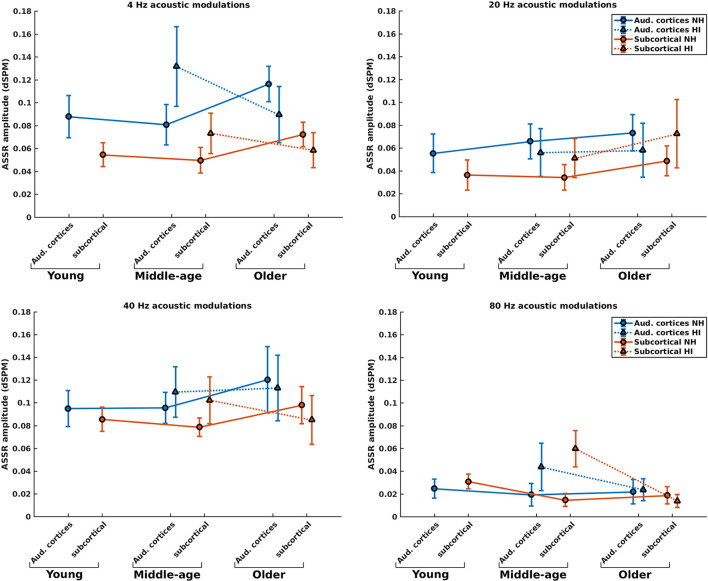
ASSR amplitudes of the neural generators in the auditory cortices and subcortical neural generators in NH and HI participants regardless of the side of stimulation across age and modulation frequency. The circle and triangle symbols indicate the pooled means (i.e., the weighted average of amplitudes across the side of stimulation and the side of generators; number of subjects as weights), and error bars represent the pooled standard deviations (69).

Figure 3 illustrates the mean response strengths for the cortical and subcortical neural generators (for anatomical locations, see Figure 2) for young, middle-aged, and older listeners for each of the four modulation frequencies. A significant main effect of HI was found in the middle-aged and older participants for 4, 20, 40, and 80 Hz modulations (see Table 1). However, the main effects in middle-aged participants were the opposite of those of older participants. For the middle-aged participants, the response strengths of listeners with HI were larger than those of listeners with NH. In contrast, for the older participants, a significantly smaller response strength was observed in the listeners with HI compared to the NH ones for 4, 40, and 80 Hz, yet not for 20 Hz acoustic modulations.

**Table 1:** The results of the main effect of hearing impairment and *post-hoc* testing for ASSR amplitude and phase coherence.

		**ASSR amplitude**		**Phase coherence**	
		**Middle-aged** **NH, HI**	**Older NH, HI**	**Middle-aged** **NH, HI**	**Older NH, HI**
4 Hz	Aud. cortices	d = −1.9 *p* <0.001	d = 1.3 *p* <0.001	d = −1.3 *p* < 0.001	d = 0.7 *p* < 0.001
	Subcortical	d = −1.6 *p* < 0.001	d = 1.0 *p < * 0.001	d = −0.6 *p* < 0.001	d = -0.3 *p* < 0.01
	Main effect	d = −1.7 *p* < 0.001	d = 1.1 *p* < 0.001	d = −0.7 *p* < 0.001	d = -0.1 n.s.
20 Hz	Aud. cortices	d = 0.5 *p* < 0.001	d = 0.7 *p* < 0.001	d = 0.9 *p* < 0.001	d = 0.6 *p* < 0.001
	Subcortical	d = −1.2 *p* < 0.001	d = -1.1 *p* < 0.001	d = −0.1 n.s.	d = -1.1 *p* < 0.001
	Main effect	d = −0.6 *p* < 0.001	d = -0.6 *p* < 0.001	d = 0.1 n.s.	d = -0.7 *p* < 0.001
40 Hz	Aud. cortices	d = −0.8 *p* < 0.001	d = 0.2 n.s.	d = 0.1 n.s.	d = 2.0 *p* < 0.001
	Subcortical	d = −1.6 *p* < 0.001	d = 0.7 *p* < 0.001	d = 1.0 *p* < 0.001	d = 1.5 *p* < 0.001
	Main effect	d = −1.3 *p* < 0.001	d = 0.5 *p* < 0.001	d = 0.8 *p* < 0.001	d = 1.7 *p* < 0.001
80 Hz	Aud. cortices	d = −1.5 *p* < 0.001	d = -0.1 n.s.	d = −1.0 *p* < 0.001	d = 0.3 n.s.
	Subcortical	d = −4.0 *p* < 0.001	d = 0.7 *p* < 0.001	d = −1.9 *p* < 0.001	d = 0.8 *p* < 0.001
	Main effect	d = −3.2 *p* < 0.001	d = 0.4 *p* < 0.001	d = −1.6 *p* < 0.001	d = 0.6 *p* < 0.001

*The post-hoc testing was performed per age cohort and modulation frequency for the neural generators in the auditory cortices and subcortical region. Cohen's d and p-value were reported for different age cohorts and different modulation frequencies. No significant differences were indicated with “n.s”*.

*Post-hoc* testing in middle-aged participants showed significantly larger response strengths for listeners with HI than NH listeners for both the cortical and subcortical neural generators and different modulation frequencies. The only exception was for the cortical generators with larger response strengths for NH than participants with HI in response to the 20 Hz stimuli. The effect sizes suggest a large difference [d ≥ 0.8; (69, 70)] between HI and NH middle-aged listeners in response to the four different modulation frequencies. The results of *post-hoc* testing are summarized in Table 1.

For the older listeners, *post-hoc* testing revealed significantly smaller response strengths for listeners with HI compared to NH participants in the subcortical category of neural generators for all modulation frequencies, except for 20 Hz. Similarly, *post-hoc* testing revealed significantly smaller response strengths for listeners with HI compared to NH participants for neural generators in the auditory cortex in response to 4 and 20 Hz acoustic stimuli. The effect sizes demonstrate a large difference (d ≥ 0.8) between HI and NH older listeners for 4 Hz and a medium difference (d ≥ 0.5) for other frequencies.

Briefly, the response strength of the listeners with HI showed two different patterns of the changes in the middle-aged and older participants for most modulation frequencies. With the middle-aged participants, the response strength of listeners with HI was larger than those of NH listeners. In contrast, significantly smaller response strengths were observed in the listeners with HI compared to the NH ones for most modulation frequencies for the older participants.

### Effect of hearing impairment on the phase coherence of the neural generators

**Figure 4:**
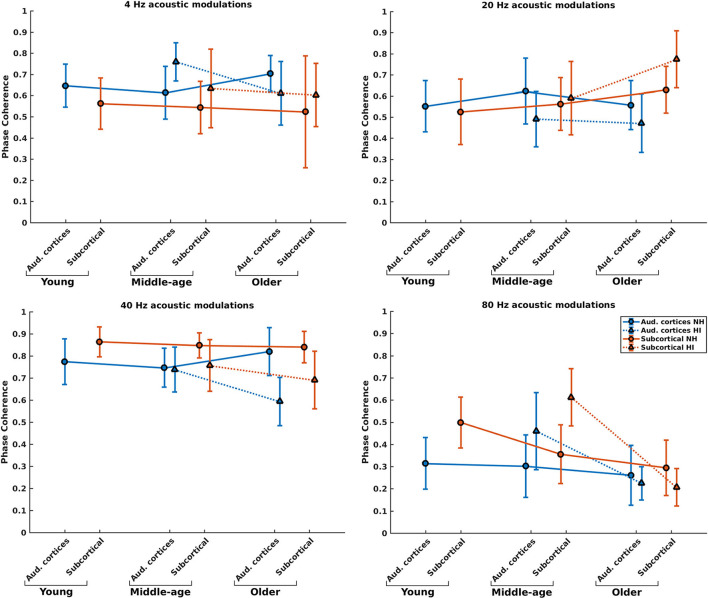
Phase coherence of the neural generators in auditory cortices and subcortical area in NH and HI participants regardless of the side of stimulation across age and modulation frequency. The circle and triangle symbols indicate the pooled means, and error bars represent the pooled standard deviations (69).

Phase coherence reflects the changes in phase-locking of the responses regardless of the strength of the responses. Figure 4 illustrates the mean phase coherence for the cortical and subcortical neural generators (for anatomical locations, see Figure 2) for young, middle-aged, and older listeners for each of the four different modulation frequencies. A significant main effect of hearing impairment was observed for the middle-aged and older participants for most of the modulation frequencies. Detailed results are summarized in Table 1. Again, two different patterns of the changes were observed in the middle-aged with HI and older participants with HI. In most of the middle-aged participants' comparisons, HI listeners' phase-locking was larger than those of NH listeners. In contrast, a significantly smaller phase-locking was observed for the older HI participants than for the older NH ones.

*Post-hoc* testing in middle-aged participants showed a significantly larger phase coherence for listeners with HI than NH listeners in the cortical and subcortical neural generators for 4 and 80 Hz amplitude-modulated stimuli. The effect sizes of mean differences (Cohen's d) in these comparisons were medium or large [d ≥ 0.5; (69, 70)]. However, there was less phase coherence in listeners with HI than NH listeners for cortical neural generators at 20 Hz and subcortical neural generators at 40 Hz stimulation conditions.

For the older listeners, *post-hoc* testing revealed significantly less phase coherence for listeners with HI compared to NH participants in the auditory cortices for all modulation frequencies, except for 80 Hz. In these modulation frequencies, Cohen's d suggests a medium or large effect size (d ≥ 0.5) of mean differences (69, 70). A similar effect was observed for the subcortical neural generators in response to 40 and 80 Hz acoustic stimuli. The effect sizes were large [d ≥ 0.8; (69, 70)].

### Hemispheric lateralization and hearing impairment

**Figure 5:**
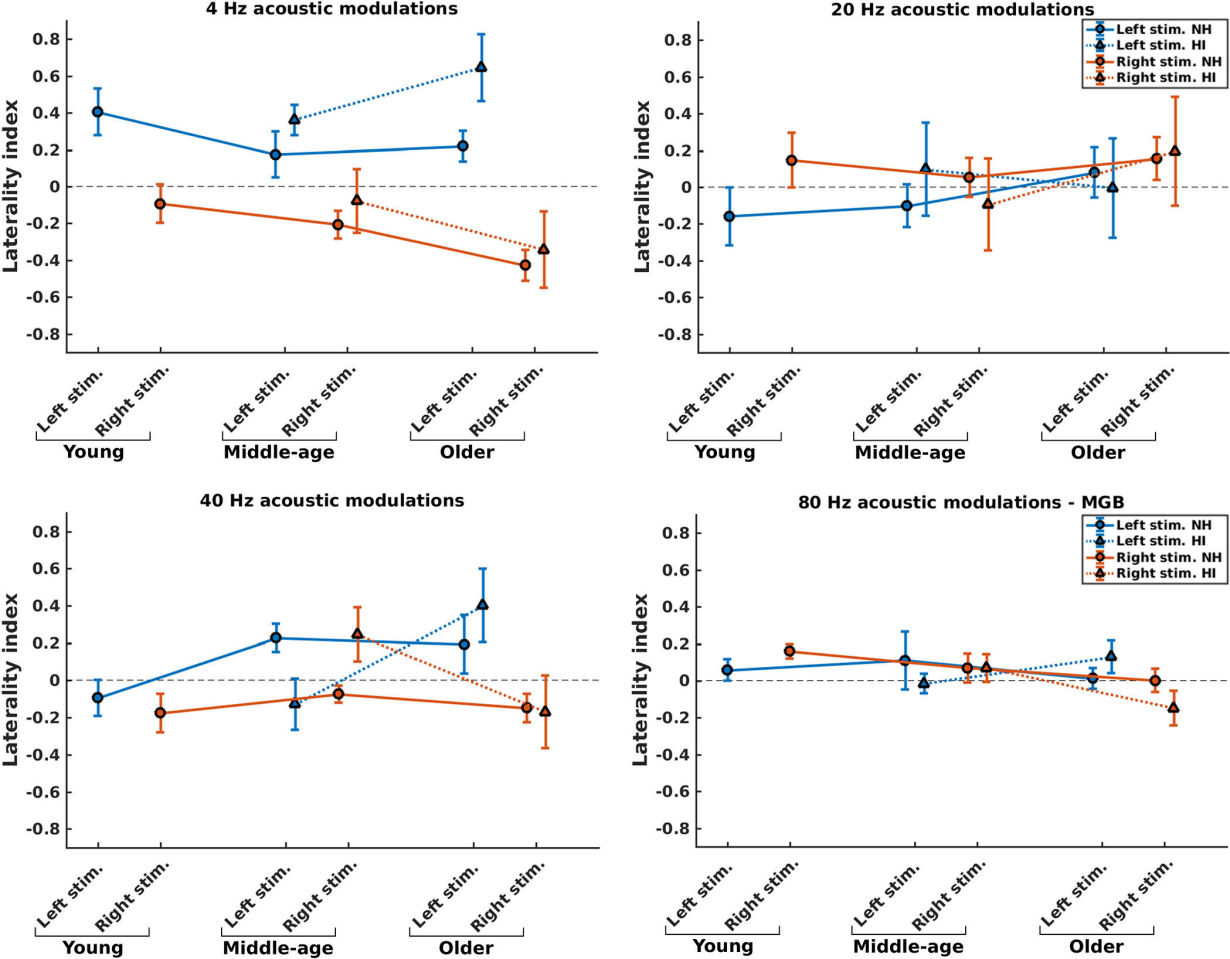
Hemispheric lateralization for normal-hearing (NH) and hearing-impaired (HI) listeners (indicated by solid lines and dotted lines, respectively) in different stimulation conditions (indicated by different colors) and different age groups. For 4, 20, and 40 Hz stimuli, the laterality indexes (LIs) were calculated based on the auditory cortex (AC), while for 80 Hz stimuli the LIs were calculated based on the medial geniculate body (MGB). The error bars illustrate the estimated standard deviations using the jackknife method (65).

To investigate potential changes in hemispheric asymmetry of envelope processing in listeners with HI and NH ones, we determined the LIs for the 4, 20, and 40 Hz modulation frequencies based on the ASSR amplitudes of the left and right auditory cortices. For 80 Hz modulation frequency, we calculated the LI based on the ASSR amplitudes of the MGB, given the importance of subcortical activities (36). Figure 5 illustrates the LIs of the AC for 4, 20, and 40 Hz ASSRs in three age groups and two sides of stimulation and the LIs of the MGB for 80 Hz ASSRs. The groups with significant hemispheric asymmetry to the left or right hemisphere were determined using a one-sample *t*-test (the results are summarized in Supplementary Table 1).

The effect of hearing impairment on hemispheric asymmetry was investigated for middle-aged and older listeners. In most stimulation conditions, the hemispheric asymmetry in the listeners with HI was significantly more toward the right hemisphere than the hemispheric asymmetry of the NH ones. More specifically, with middle-aged participants, the LIs of listeners with HI were significantly more positive (toward the right hemisphere) than those of the NH ones for the 4 Hz (both sides of stimulation), 20 Hz (left side of stimulation), and 40 Hz (right side of stimulation) modulation frequencies. However, for 80 Hz AM stimuli, the hemispheric asymmetry was less or similar for the listeners with HI than for the NH ones for the left and right sides of stimulation, respectively. In these comparisons, Cohen's d suggests a large effect size (d ≥ 0.8) of mean differences (69, 70). The results of statistical tests are summarized in Table 2.

**Table 2:** The results of the statistical comparison between the laterality index of normal-hearing (NH) and hearing-impaired (HI) listeners in different stimulation conditions.

**Stimulation condition**		**Middle-aged NH, HI**	**Older** **NH, HI**
4 Hz	Left ear	d = −1.7 *p* < 0.001	d = -3.2 *p* < 0.001
	Right ear	d = −1.1 *p* < 0.01	d = -0.5 n.s.
20 Hz	Left ear	d = −1.1 *p* < 0.01	d = 0.4 n.s.
	Right ear	d = 0.8 n.s.	d = -0.1 n.s.
40 Hz	Left ear	d = 3.3 *p* < 0.001	d = -1.2 *p* < 0.05
	Right ear	d = −3.1 *p* < 0.001	d = 0.1 n.s.
80 Hz	Left ear	d = 1.0 *p* < 0.05	d = -1.5 *p* < 0.001
	Right ear	d = −0.1 n.s.	d = 1.9 *p* < 0.001

For the older participants, the LIs of listeners with HI were similar or significantly more positive (toward the right hemisphere) than those of the NH ones for the 4, 20, and 40 Hz modulation frequencies for both the left and the right sides of stimulation. A similar effect was observed for the 80 Hz modulations presented to the left ear, while for the right side of stimulation, the LI of the listener with HI is more negative (toward the left hemisphere) than that of the NH group. In these comparisons, the effect sizes were large [d ≥ 0.8; (69, 70)].

## Discussion

### Effect of age-related hearing impairment on the dynamics of neural generators

Our results indicated meaningful changes in the neural dynamics of middle-aged and older listeners with HI compared to those of middle-aged and older NH listeners. The effect of hearing impairment on the dynamics of the cortical and subcortical neural generators was investigated in persons with no indication of mild cognitive impairment to avoid the confounding factors of age and cognitive ability as much as possible. The acoustic modulations were presented at equal loudness levels to the participants with HI to correct for stimulus audibility. The cortical and subcortical neural generators' activity was reconstructed using the MNI approach. It should be noted that the selected parameters in the MNI approach, such as the number of layers of the head model, the conductivity of brain tissues, and the regularization parameters, may influence the results of the source reconstruction. Since the same methods and parameters were used for the different age cohorts with and without hearing impairment, the comparisons and the conclusions drawn from them remain reasonable.

Two different patterns of alterations were observed in the middle-aged participants with HI and older participants with HI. For middle-aged participants, we mainly found enhanced response strength and higher phase-locking in the HI group than NH, while for the older ones, we found decreased response strength and less phase-locking in the listeners with HI. The findings of middle-aged people agree with the literature (6, 21, 22). However, our results for the older participants are novel and different from sensor-level analysis on the same data as here (11). These findings for middle-aged and older participants with HI are elaborated on below.

Our observation of enhanced response strength in HI middle-aged listeners' auditory cortex follows Millman et al. (22) and Fuglsang et al. (21). Millman and colleagues investigated the neural synchronizations in response to 2 Hz acoustic modulated noise between HI and NH similarly aged persons (~60 years old). Fuglsang et al. (21) reported magnified cortical responses in participants with HI compared to NH participants for tone sequences modulated at slow rates (4 Hz) during a passive listening task. They had also corrected for the audibility of auditory stimuli for the participants with HI, and the age range of participants was similar (~65 years old).

The enhanced neural responses in the subcortical generators of middle-aged adults with HI are in line with animal studies which have shown that peripheral hearing loss is associated with increased neural responses to amplitude-modulated stimuli in the auditory nerve fibers (6–8) and the midbrain (9). Similarly, human electrophysiological studies reported enhanced neural responses in the brainstem of adults around 60 years old with HI relative to NH ones in the same age range (10, 11).

Only a few studies report how age-related hearing loss affects temporal envelope processing in older people (70–80 years old). Using source analysis, we observed significantly less response strength for the older adults with HI than the NH ones. However, sensor-level analysis on the same data yielded no significant difference in response strengths between the older adults with HI and NH ones (11). Note that the response strengths in the sensor-level reflect a weighted average of the activity (due to the volume conduction). Therefore, this approach may not be as sensitive to small changes as brain source analysis which estimates the original neural activity of each generator.

The reduced neural synchronization (response strength and phase-locking) in the older adults with HI in the current study agrees with the observations of Hao et al. (71). They found reduced frequency-following responses (FFRs), under quiet and noise conditions, in the older adults with presbycusis (60–82 years old) compared to NH similarly aged persons. However, data regarding the effect of hearing impairment on FFRs are not very consistent [for review, see (72)]. For instance, Presacco et al. (73) did not find significant differences between the FFRs in the older adults with HI (average 71 years old) and those in the NH adults (average 65 years old). The discrepancies between the findings of different FFR studies could be due to the different age ranges involved.

In an experiment using continuous speech, Decruy et al. (74) found evidence of enhanced envelope tracking to the target talker in older adults with HI compared to NH listeners. In a similar experiment, Presacco et al. (73) found no differences. These results are different from our findings in the older participants with HI. The first possible reason could be differences between experimental conditions. The envelope tracking in our experiment is unattended, while in the experiment of Decruy et al. (74), the participant should attend to the stimuli. In speech envelope tracking onset responses play an important role, while it is not applicable for ASSRs. The second reason for different results refers to the source-level analysis in our study and reconstructing the activity of neural generators along the auditory pathway, while Decruy et al. (74) and Presacco et al. (73) used sensor-level analysis which considers all cortical activities.

For the relatively low frequencies (below 50 Hz), there is an age-related enhancement in the neural responses of NH older adults compared to those of young and middle-aged adults (19). Considering the age-related enhancement in the NH older adults and the enhancement effect in the middle-aged adults with HI (the current study), we expected to find an aggravated effect of hearing impairment in the older participants with HI. However, our results for the older adults with HI showed reduced responses compared to NH participants in the same age cohort. This novel finding suggests that the reduced effect of age-related hearing loss and age-related degradation in the older cohort (70–80 years) may be greater than a compensatory enhancement effect in the representation of envelope processing in this age cohort.

### Potential mechanisms underlying the changes in temporal envelope processing

Homeostatic compensatory mechanisms can explain the enhanced response strength and phase-locking in the middle-aged adults with HI. It is known that diminished cochlear output in adults with HI activates various mechanisms which induce central gain to increase neural excitability (75–77). However, the potential compensatory mechanisms could be considered maladaptive, because the response strength and phase-locking in the middle-aged adults with HI were even higher than those of NH middle-aged listeners.

For example, the hearing-impaired auditory nerve fibers at the subcortical level show steeper loudness growth than NH ones (7, 78) and enhanced onset responses (79). Spontaneous activity is enhanced in the inferior colliculus (80) and the auditory cortex of older compared to young animals (75, 81, 82). Along the auditory pathway (from the brainstem up to the cortex), the influx of inhibitory neurotransmitters into excitatory neurons decreases, while it is preserved for inhibitory neurons (83–85).

The reduced response strength in the older adults with HI can be explained by the normal age-related changes in this age cohort. In a previous study on the adults with normal audiometric thresholds, we observed enhanced neural responses to envelope modulations for NH older persons compared to young and middle-aged NH individuals (19). This age-related enhancement can be attributed to the loss of functional inhibition in older adults as a compensatory mechanism (19, 86, 87). These mechanisms are used in normal-hearing older persons. On top of it, hearing impairment impacts neural processing in the older adults with HI. Consequently, the reduced response strength is detected for hearing impairment at an older age despite correcting for audibility.

Both middle-aged with HI and older adults with HI have similar patterns of hearing loss, with no significant differences in pure-tone average (PTA) across all audiometric thresholds (0.25–8 kHz) (88). However, age-related structural changes, such as cerebral atrophy and demyelination, increase with age (89, 90). The animal study of Wang et al. (91) showed that, in addition to known cochlear synaptopathy, the central synapses of spiral ganglion neurons are also pathologically changed during aging, which suggests a central synaptopathy. This central synaptopathy plays a significant role in weakened auditory input and altered central auditory processing during age-related hearing loss (91). The above-mentioned could also explain the different results for middle-aged and older adults.

### Hemispheric asymmetry

Generally, our results suggest that hearing impairment is associated with altered hemispheric asymmetry in auditory temporal processing. In most cases, this alteration occurs through shifting toward the right hemisphere. This observation follows previous studies suggesting altered hemispheric asymmetry of event-related potentials in older adults with HI (32, 92).

To the best of our knowledge, this study is one of the first to investigate the association between hearing impairment and hemispheric asymmetry in temporal envelope processing using source analysis. In line with the HAROLD model (31), it was previously documented that hemispheric asymmetry for temporal envelope processing is reduced (more symmetric) for the NH older adults compared to those of the younger ones (29, 30). Using source analysis, Farahani et al. (19) reported that hemispheric asymmetry is reduced in NH older adults compared to NH younger ones in response to the 20 and 80 Hz amplitude-modulated stimuli. Although NH older is thus expected to be associated with less asymmetrical neural processing, our older participants with HI exhibit asymmetrical processing patterns. The LI in the middle-aged and older participants with HI exhibits a hemispheric asymmetry more toward the right hemisphere than the hemispheric asymmetry of the NH ones. This novel observation may be explained by the reduced integrity of white matter tracts related to presbycusis (33). The corpus callosum is a large bundle of white matter tracts that play a key role in interhemispheric interactions (93). As such, white matter deficits in people with severe age-related hearing loss can impact the hemispheric asymmetry in temporal envelope processing. However, further research is needed to clarify the relationship between the changes in the white matter and the altered hemispheric asymmetry in older adults with HI.

### The role of source-level analysis

In electrophysiological measurements, the recorded data at each sensor are a weighted average of the activity of several neural generators due to the volume conduction of the brain tissue. However, brain source analysis allows us to estimate the original activity of each neural generator. Such an analysis increases our understanding of the potential alterations at different levels of the auditory pathway across age and with or without hearing impairment.

Furthermore, brain source analysis enables us to detect relatively small changes in the activity of a neural generator which may not be detectable in the sensor-level analysis. For example, values of Cohen's d (ASSR amplitude, Table 1) suggest that the differences in the responses between listeners with HI and NH are larger than those between HI and NH older adults. In middle-aged adults, the results of sensor-level analyses (i.e., enhanced response strengths in listeners with HI, 10) were in line with the results of source-level analysis (i.e., the current study). However, in older adults, where the differences are smaller, the sensor-level analysis yielded no significant difference in response strengths between the older adults with HI and NH ones (11), while brain source analysis using MNI on the same data revealed significant changes for the neural generators.

## Conclusion

The present study investigated the effect of age-related hearing loss on the dynamics of the neural generators involved in the temporal envelope processing for middle-aged and older adults. The activity of the cortical and subcortical neural generators of ASSRs was reconstructed for participants with HI and NH ones using the MNI approach. This approach allows for a detailed analysis of the neural generators' activity along the auditory pathway (25). Our results showed that age-related hearing loss, with correction for audibility, is accompanied by changes in response strength and phase-locking of the neural generators of the ASSRs. However, the patterns of the changes in the middle-aged participants are different from those of older ones. With the middle-aged participants, the response strength and phase coherence of listeners with HI were larger than those of NH listeners. In contrast, for the older participants, a significantly smaller response strength and phase coherence were observed in the listeners with HI compared to the NH ones for most modulation frequencies. This is an essential finding to develop rehabilitation strategies for hearing-impaired persons across the aging life span.

With our novel approach, we observed that middle-aged and older participants with HI exhibit a hemispheric asymmetry more toward the right hemisphere than the hemispheric asymmetry of the NH ones. This observation can be explained by the brain structural changes associated with presbycusis in the middle-aged and older adults.

## Data availability statement

The datasets presented in this article are not readily available because of ethical and privacy restrictions. Requests to access the datasets should be directed to Astrid van Wieringen, astrid.vanwieringen@kuleuven.be.

## Ethics statement

The studies involving human participants were reviewed and approved by Medical Ethical Committee of the University Hospitals and University of Leuven. The patients/participants provided their written informed consent to participate in this study.

## Author contributions

EF, JW, and AW designed the study, contributed to the interpretation of the results, and critically revised the manuscript. EF analyzed data, performed statistical analyses, and wrote the manuscript draft. JW and AW verified the analytical methods and supported data analysis. All authors contributed to the article and approved the submitted version.

## Funding

This work was supported by the Research Council, KU Leuven, through projects C14/19/110, C14/17/046, and by the Research Foundation Flanders through FWO-projects G066213 and G0A9115. This work was also partly funded by Flanders Innovation & Entrepreneurship through the VLAIO research grant HBC.20192373.

## Conflict of interest

The authors declare that the research was conducted in the absence of any commercial or financial relationships that could be construed as a potential conflict of interest.

## Publisher's note

All claims expressed in this article are solely those of the authors and do not necessarily represent those of their affiliated organizations, or those of the publisher, the editors and the reviewers. Any product that may be evaluated in this article, or claim that may be made by its manufacturer, is not guaranteed or endorsed by the publisher.
