# Supplementary material for: Age-related hearing loss is associated with alterations in temporal envelope processing in different neural generators along the auditory pathway
Source: Front Neurol. 2022 Aug 5;13:905017. doi: 10.3389/fneur.2022.905017 (PMC9389009; doi:10.3389/fneur.2022.905017)
Supplement: Supplementary file 1 [file Table_1.DOCX]

***Supplementary materials***

**Supplementary Table 1.** The results of the tests for hemispheric lateralization of the neural source in the auditory cortex (AC) in different age cohorts (Y: young, M: middle-age, O: older) and different hearing groups (NH: normal hearing, HI: hearing impaired). The results were reported for different stimulation conditions (4, 20, 40, and 80 Hz AM stimuli presented to the left (L) or right (R) ear). N.S. indicates a non-significant differences. The results were corrected for multiple comparisons by means of the FDR method.

|  |  | **4 Hz** |  | **20 Hz** |  | **40 Hz** |  | **80 Hz*** |  |
| --- | --- | --- | --- | --- | --- | --- | --- | --- | --- |
|  |  | **L** | **R** | **L** | **R** | **L** | **R** | **L** | **R** |
| **Y** | **NH** | t(17)=13.45  p < 0.001 | t(17)=-3.82  p=0.001 | t(17)=-4.27  p < 0.001 | t(17)=4.18  p < 0.001 | t(16)=-4.09  p < 0.001 | t(17)=-7.23  p < 0.001 | t(17)=4.13  p<0.001 | t(16)=16.93  p<0.001 |
| **M** | **NH** | t(17)=5.87  p < 0.001 | t(17)=-11.83  p<0.001 | t(17)=-3.72  p < 0.01 | N.S. | t(15)=12.13  p < 0.001 | t(17)=-6.75  p < 0.001 | t(16)=2.88  p<0.05 | t(17)=3.66  p<0.01 |
|  | **HI** | t(12)=16.08  p < 0.001 | N.S. | N.S. | N.S. | t(11)=-3.23  p < 0.01 | t(12)=6.06  p < 0.001 | N.S. | t(10)=3.05  p<0.05 |
| **O** | **NH** | t(14)=10.23  p < 0.001 | t(15)=-20.62  p < 0.001 | N.S. | t(15)=5.37  p < 0.001 | t(13)=4.55  p < 0.001 | t(14)=-7.46  p < 0.001 | N.S. | N.S. |
|  | **HI** | t(10)=11.85  p < 0.001 | t(10)=-5.51  p < 0.001 | N.S. | N.S. | t(6)=5.41  p < 0.01 | t(7)=-2.46  p < 0.05 | t(10)=4.79  p<0.001 | t(10)=-5.3  p<0.001 |

*Note: For 80 Hz modulation frequency we calculated the LI based on the ASSR amplitudes of the MGB.
